# Risk Factors for 30-day Mortality in Patients with Surgically Treated Necrotizing Enterocolitis: A Multicenter Retrospective Cohort Study

**DOI:** 10.1055/a-2536-4757

**Published:** 2025-03-21

**Authors:** Adinda G.H. Pijpers, Ceren Imren, Otis C. van Varsseveld, Laurens D. Eeftinck Schattenkerk, Claudia M.G. Keyzer-Dekker, Jan B.F. Hulscher, Elisabeth M.W. Kooi, Chris H.P. van den Akker, Joost van Schuppen, H. Rob Taal, Jos W.R. Twisk, Joep P.M. Derikx, Marijn J. Vermeulen

**Affiliations:** 1 Department of Pediatric Surgery, Emma Children's Hospital, Amsterdam UMC, University of Amsterdam and Vrije Universiteit Amsterdam, Amsterdam, The Netherlands; 2 Amsterdam Reproduction and Development Research Institute, Amsterdam UMC, University of Amsterdam, Amsterdam, The Netherlands; 3 Amsterdam Reproduction and Development Research Institute, University of Amsterdam, Amsterdam, The Netherlands; 4 Department of Pediatric Surgery, Sophia Children's Hospital, Erasmus MC, Rotterdam, The Netherlands; 5 Department of Surgery, Division of Pediatric Surgery, Beatrix Children's Hospital, University Medical Center Groningen, Groningen, The Netherlands; 6 Department of Pediatrics, Division of Neonatology, Beatrix Children's Hospital, University Medical Center Groningen, Groningen, The Netherlands; 7 Department of Pediatrics, Neonatology, Emma Children's Hospital, Amsterdam UMC, University of Amsterdam and Vrije Universiteit Amsterdam, Amsterdam, The Netherlands; 8 Department of Radiology and Nuclear Medicine, Emma Children's Hospital, Amsterdam UMC, University of Amsterdam and Vrije Universiteit Amsterdam, Amsterdam, The Netherlands; 9 Department of Neonatal and Paediatric Intensive Care, Division of Neonatology, Erasmus University Medical Center, Rotterdam, The Netherlands; 10 Department of Epidemiology and Biostatistics, VU University Medical Centre, Amsterdam, The Netherlands

**Keywords:** NEC totalis, portal venous gas, neonates, cardiovascular support

## Abstract

**Purpose:**

Necrotizing enterocolitis (NEC) is a leading cause of death in very preterm born infants. The most severe variant is NEC totalis (NEC-T), where necrosis of the small intestines is so extensive that curative care is often withdrawn. Mortality and NEC-T are difficult to predict before surgery, complicating counseling and decision-making. This study's aim was to identify preoperative risk factors for overall 30-day mortality and NEC-T in preterm born infants with surgical NEC.

**Methods:**

This multicenter retrospective cohort study included preterm born infants (<35 weeks) surgically treated for NEC between 2008 and 2022. NEC-T was defined as necrosis of the majority of small intestine, leading to a surgical open-close procedure without curative treatment. Preoperative risk factors for 30-day postoperative mortality, NEC-T, and mortality without NEC-T were assessed using multivariable logistic regression analyses.

**Results:**

Among the 401 patients included, the 30-day mortality rate was 34.2% (137/401), of which 18.7% (75/401) involved NEC-T. Significant risk factors for mortality were male sex (odds ratio [OR]: 2.53; 95% confidence interval [CI]: 1.54–4.16), lower birthweight (OR: 0.91; 95% CI: 0.86–0.96/100 g), portal venous gas (PVG) on abdominal radiograph (OR: 1.89; 95% CI: 1.11–3.20), need for cardiovascular support between NEC diagnosis and surgery (OR: 3.26; 95% CI: 2.02–5.24), and shorter time between diagnosis and surgery (OR: 0.74; 95% CI: 0.65–0.84). Similar risk factors were found for NEC-T. In patients without NEC-T, the need for cardiovascular support (OR: 2.33; 95% CI: 1.33–4.09) and time between diagnosis and surgery (OR: 0.77; 95% CI: 0.64–0.91) were significant.

**Conclusion:**

Male sex, lower birthweight, PVG, cardiovascular support, and a short interval between NEC diagnosis and surgery are preoperative risk factors for 30-day mortality and NEC-T. Preoperative cardiovascular support and a shorter time interval between diagnosis and surgery are also risk factors for mortality without NEC-T.

**Level of evidence:**

II

## Introduction

Necrotizing enterocolitis (NEC) is a severe gastrointestinal disease primarily affecting preterm and low birthweight infants. The global incidence among very low birthweight infants (<1,500 g) varies across countries and centers but is estimated to be around 7% according to a recent systematic review. 1 Inflammatory responses in the immature intestinal tract lead to ischemia, often followed by necrosis and bowel perforation. Despite medical treatments, including antibiotics, nil-per-mouth, parenteral nutrition, and analgesics, surgical intervention is necessary in up to 60% of patients with confirmed NEC to resect the affected intestinal segments. 2 3 In 11 to 15% of all NEC cases, the necrosis of the small intestine is so extensive that no curative treatment is considered possible. 4 5 This severe form known as NEC totalis (NEC-T) is difficult to predict as it only becomes apparent during laparotomy. In such cases, the medical team perioperatively often decides to perform an open-close procedure and to provide palliative care, leading to a fatal outcome.

Predicting the clinical course and short-term outcome of NEC is challenging due to the varying courses of onset, recovery, or deterioration despite intensive nonsurgical treatment. The prognosis is also difficult to predict since clinical signs often do not accurately reflect the extent of intestinal necrosis until surgical exploration. 6 Various risk factors for mortality among general NEC patients have been identified, including male sex, low birthweight, and hypotension requiring cardiovascular support, but their association with outcomes in surgically treated NEC remains uncertain. 7 8 9 10 Specific short-term risk factors for the more severe cases of surgically treated NEC and NEC-T have not been thoroughly described in the existing literature. Understanding these prognostic factors is crucial for preoperatively counseling of parents. This study aimed to identify preoperative risk factors for short-term postoperative mortality, with or without NEC-T, in a cohort of preterm infants with surgically treated NEC.

## Methods

### Study Design and Population

We conducted a retrospective multicenter cohort study involving three NICUs of those with the highest level of neonatal intensive care and pediatric surgery available in the Netherlands: the Emma Children's Hospital of Amsterdam University Medical Centers (AUMC), the Erasmus University Medical Center Sophia Children's Hospital (EMC), and the University Medical Center Groningen (UMCG). In the Netherlands, intensive care treatment is offered starting from 24 weeks of gestation, after careful prenatal counseling. All centers followed the 2014 Dutch neonatal recommendations on NEC treatment. 11

As this retrospective study was not subject to the Dutch Medical Research Involving Human Subjects Act, research ethics approval was not required. In the AUMC, a general consent opt-out approach was used (W18_233#18.278). In the EMC and UMCG, informed consent requirements were waived (MEC-2013-409 and CTc 201800771, respectively) based on the observational nature of the study.

The study included all patients born <35 weeks' gestational age (GA) between 2008 and 2022 who underwent surgery for NEC in any of the three participating centers. Exclusion criteria were surgery for stenosis after medical NEC treatment and gastrointestinal surgery unrelated to NEC (such as focal intestinal perforation or milk curd syndrome). Four physicians (L.D.E.S., O.C.V., C.I., A.G.H.P.) independently assessed cases for inclusion and exclusion criteria at their sites and double-checked ≥10% of other sites' data. At each participating site, disagreements were resolved through consensus meetings involving local neonatologists (C.H.v.d.A., M.J.V., E.M.W.K.) and pediatric surgeons (J.P.M.D., C.M.G.K., J.B.F.H.).

### Data Collection and Definitions

The following data were extracted from individual electronic patient records: sex, GA, birthweight, birthweight Z-score, 12 antenatal steroids, mode of delivery (vaginal or cesarean section), multiple births, minor and major congenital heart defects, intraventricular hemorrhage (no, grade 1–2, and grade 3 or higher), 13 hypotension requiring cardiovascular support during the first week of life and/or support between NEC onset and surgery. Reports of preoperative radiological images were screened for the presence of pneumatosis intestinalis, portal venous gas (PVG), pneumoperitoneum, and ascites. Due to the retrospective design of this study, only the radiological reports were evaluated, without routine reassessment of the radiological images. The surgical decision-making at the time was based on consensus within the medical team, including neonatologists and pediatric surgeons, and in some cases neurologists, anesthesiologists, and medical ethicists. The indication for surgery was based on findings such as pneumoperitoneum or clinical deterioration despite maximal medical treatment. The information regarding the indication for surgery was retrieved from the patient's electronic health records. Information on the surgical procedure, time between NEC onset and surgery, extent of intestinal necrosis, and intraoperative clinical decisions were collected from the surgical reports. The definition of NEC was based on a combination of clinical signs and symptoms, radiological signs, and perioperative aspect of the intestinal tissue, corresponding with Bell's classification grade II and III. 14 NEC diagnosis was confirmed by pathology reports of surgically resected tissue.

The day of NEC onset was defined as the first day the diagnosis NEC stage II/III was made and medical NEC therapy was initiated. Cases of focal intestinal perforation, defined as isolated perforation in a normal-appearing bowel without features of NEC such as pneumatosis intestinalis or necrosis, were not classified as NEC. 15 The 30-day postoperative mortality was defined as all-cause death within 30 days after primary surgery. NEC-T was defined as the condition in which at least the majority of the small intestine was necrotic, leading surgeons and neonatologists to determine that no further meaningful treatment options were available, taking the prognoses of the patient's other medical conditions also into consideration. This led to an open-close procedure followed by palliative care. 16 Major congenital heart defects were defined as cardiac anomalies requiring cardiothoracic surgery, except for ductus arteriosus closure. Minor cardiac birth defects included all nonsurgical cardiac anomalies, except persistent foramen ovale, persistent ductus arteriosus, atrial septal defect, and dextrocardia, which are considered mild forms. 17 As these are mild and generally have limited clinical consequences, they were not analyzed in detail and fall beyond the scope of this article. Multiorgan failure was defined as the presence of altered organ function in an acutely ill patient, such that homeostasis cannot be maintained without intervention, involving two or more organ systems. 18

### Statistical Analysis

Normally distributed variables are reported as mean ± standard deviation, whereas non-normally distributed variables are reported as median with interquartile range (IQR). All analyses were conducted using SPSS software version 28.0 (IBM SPSS Statistics for Windows, Armonk, New York, United States). A *p* -value < 0.05 was considered statistically significant.

Potential risk factors were selected based on available literature and a directed acyclic graph. 19 To identify potential risk factors for 30-day mortality, NEC-T and mortality without NEC-T, univariable logistic regression analyses were performed. Selection of the variables to be included in the analyses was based on low collinearity, defined as a correlation coefficient with other variables <0.8. The input variables were sex, birthweight, multiple births, mode of delivery, presence of cardiac anomaly (minor and major), time (in days) between NEC onset and surgery, pneumatosis intestinalis, PVG, pneumoperitoneum, and need for cardiovascular support between NEC onset and surgery. Variables with a *p* -value of <0.05 in the univariable analysis were included in the multivariable analysis. Data on these variables were 99,8% complete and were not imputed (complete case analysis).

Three multivariable logistic regression models were constructed to assess 30-day mortality: (1) for the total population, (2) for patients with NEC-T, and (3) for patients without NEC-T. For the three models, first univariable logistic regression analyses were performed. Secondly, backward Wald selection was applied for manual selection of variables for the final multivariable models. Results are reported as odds ratios (ORs) with 95% confidence intervals (95% CI). The Nagelkerke R-square was calculated as an indicator of the quality of the final multivariable models. Additionally, a receiver operating characteristic (ROC) curve was created to assess model performance. The area under the curve (AUC) was used to quantify the overall discriminative quality of the model, with an AUC score of 0.50 to 0.69 considered poor, 0.70 to 0.79 moderate, and ≥0.8 excellent.

## Results

### Patient Characteristics

A total of 401 patients who underwent surgical treatment for NEC were included in this study. Baseline characteristics are presented in Table 1 . More than half (59.9%) were male. Median GA was 27 ^+5^ weeks (IQR: 26 ^+0^ –30 ^+6^ ), median birthweight was 1,029 grams (IQR: 800–1,412), and median Fenton Z-score was 0.0 (IQR: −0.6 to 0.6). Nearly half (47.1%) were delivered via cesarean section. A minor congenital heart defect was confirmed in 23 (5.7%) and a major congenital heart defect in four patients (1.0%). Cardiovascular support was provided to 73 patients (18.4%) in the first week of life and to 174 (43.8%) between NEC onset and surgery. Median postnatal age at NEC onset was 10 days (IQR: 7.0–19.0). Radiological imaging showed pneumatosis intestinalis in 308 patients (76.8%). Signs of pneumoperitoneum were found in 201 (50.1%), PVG in 105 (26.2%), and ascites in 40 patients (10.0%). The median time between NEC onset and surgery was 1 day (IQR: 0.0–3.0), with 58.4% of patients undergoing surgery within 1 day of onset. The median age at surgery was 11.0 days of life (IQR: 8.0–22.0). The surgeries after bowel resection involved primary anastomosis (20.2%), stoma creation (51.6%), or a combination of both (7.2%). NEC-T was observed in 75 patients (18.7%), leading to an open-and-close procedure in the best interest of the patient because curative treatment was deemed futile. With parental consent, these patients were given comfort care and deceased. Intraoperative characteristics of patients without NEC-T are shown in Supplementary Appendix A (available in the online version).

**Table 1:** Baseline characteristics

Patient characteristics a		Total *N* = 401	30-day mortality *N* = 137	NEC-T *N* = 75	Total missing
Male		240 (59.9)	95 (69.3)	53 (70.7)	0
Gestational age, wk		27.0 (26.0–30.5)	27.0 (25.0–28.0)	26.0 (25.0–28.0)	0
Birthweight, g		1,029 (800–1,412)	950 (752–1,170)	940 (715–1,130)	0
Fenton Z-score		0.0 (−0.6 to 0.6)	0.0 (−0.6 to 0.5)	−0.1 (−0.6 to 0.6)	0
Postnatal age at diagnosis, d		10 (7.0–19.0)	10.0 (7.0–17.5)	9.0 (7.0–16.0)	0
Mode of delivery, cesarean section		189 (47.1)	64 (46.7)	36 (48.0)	0
Multiple births		121 (30.2)	44 (32.1)	25 (33.3)	0
Time between diagnosis and surgery, d		1.0 (0.0–3.0)	1.0 (0.0–1.0)	1.0 (0.0–1.0)	0
Antenatal steroids	No administration Partially (1 dose) Complete (2 doses)	77 (22.4) 55 (16.0) 212 (61.6)	22 (18.0) 20 (16.4) 80 (65.6)	6 (9.2) 12 (18.5) 47 (72.3)	57
Cardiac anomaly	Minor anomaly with PDA Minor anomaly without PDA Major cardiac anomaly	167 (41.6) 23 (5.7) 4 (1.0)	59 (43.1) 5 (3.6) 1 (0.7)	34 (45.3) 4 (5.3) 1 (1.3)	0
IVH	No IVH Grade 1–2 Grade 3 and higher	292 (73.0) 96 (23.9) 12 (3.0)	95 (69.9) 36 (26.4) 5 (3.7)	62 (82.7) 13 (17.3) 0 (0.0)	0
Cardiovascular support in the first week of life		73 (18.4)	42 (30.7)	24 (32.0)	5
Cardiovascular support between NEC diagnosis and surgery		174 (43.8)	85 (62.0)	47 (62.7)	4
Radiological findings	Pneumatosis intestinalis Pneumoperitoneum Portal venous gas Ascites	308 (76.8) 201 (50.1) 105 (26.2) 40 (10.0)	108 (78.8) 67 (48.9) 46 (33.6) 10 (7.3)	60 (80.0) 33 (44.0) 30 (40.0) 5 (6.7)	0 0 0 0
Age at surgery, d		11.0 (8.0–22.0)	11.0 (7.0–21.0)	10.0 (7.0–18.0)	1
Mode of treatment	Primary anastomosis Stoma creation Anastomosis and stoma Peritoneal drainage Peritoneal drainage and stoma Overstitching No resection No treatment (open–close)	81 (20.2) 207 (51.6) 29 (7.2) 1 (0.2) 4 (1.0) 1 (0.2) 3 (0.7) 75 (18.7)	10 (7.3) 44 (32.4) 7 (5.1) 0 (0.0) 1 (0.7) 0 (0.0) 0 (0.0) 75 (55.1)	– – – – – – – 75 (100.0)	2
Total hospital stay, d		40.0 (15.5–82.5)	11.0 (7.0–23.0)	10.0 (6.0–16.0)	0
Time between surgery and death, d		1.0 (0.0–14.5)	1.0 (0.0–3.0)	0.0 (0.0–1.0)	0
Postnatal age at death, d		17.0 (10.0–43.8)	13.0 (9.0–26.5)	10.0 (7.0–18.0)	0

Abbreviations: IVH, intraventricular hemorrhage; NEC, necrotizing enterocolitis; NEC-T, necrotizing enterocolitis-totalis; PDA, persistent ductus arteriosus.

a Data presented as number (%) or median (interquartile range).

### 30-day Postoperative Mortality

A total of 137 patients (34.2%) died within 30 days after surgery, including the 75 (54.7%) in whom NEC-T was observed. Death in the 62 patients (45.3%) without NEC-T was related to ongoing sepsis, therapy-resistant multiorgan failure (mostly cardiorespiratory failure), deterioration of neurological status, and/or ongoing NEC with extensive necrosis of small intestines in redo surgery, and all received comfort care after surgery. The median age at death was 17.0 days (IQR: 10.0–43.8) and the median time elapsed between surgery and death was 1 day (IQR: 0.0–14.5).

### Risk Factors for 30-day Overall Postoperative Mortality

**Fig. 1:**
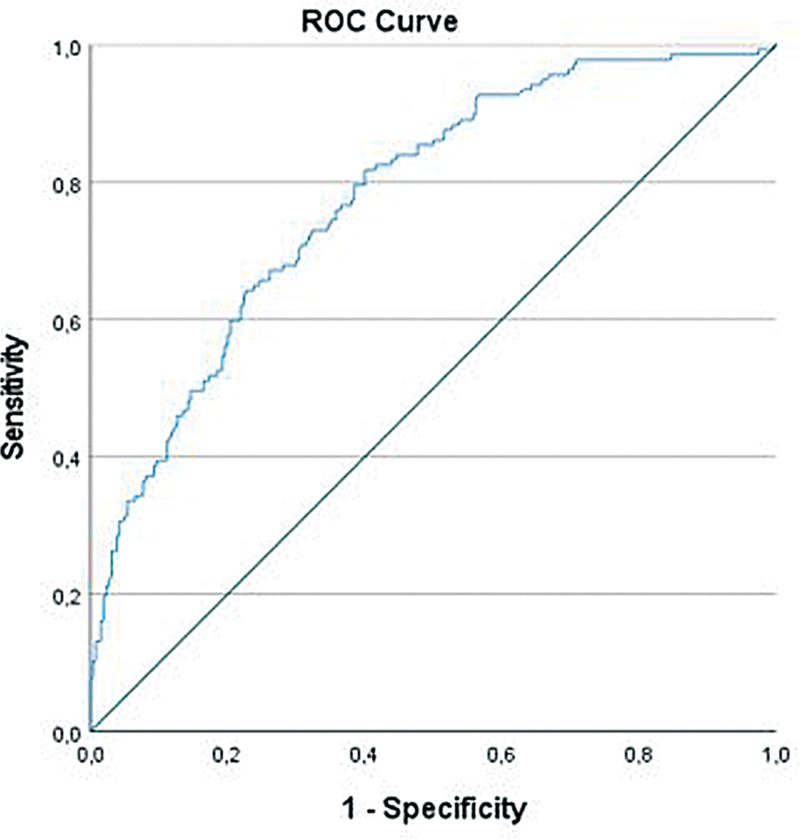
ROC curve of predicted probabilities for 30-day mortality after surgery for NEC. NEC, necrotizing enterocolitis; ROC, receiver operating characteristics. Area under the curve: 0.77.

Univariable logistic regression showed that male sex, birthweight, need for cardiovascular support between NEC onset and surgery, PVG in radiological reports, and time from onset to surgery (in days) were significantly related to 30-day postoperative mortality ( Table 2 ). These parameters were subsequently included in the multivariable analysis. Multivariable logistic regression showed that male sex (OR: 2.53; 95% CI: 1.54–4.16), birthweight (OR: 0.91; 95% CI: 0.86–0.96 per 100 g), signs of PVG on radiological imaging (OR: 1.89; 95% CI: 1.11–3.20), need for cardiovascular support between NEC onset and surgery (OR: 3.26; 95% CI: 2.02–5.24), and time between onset and surgery in days (OR: 0.74; 95% CI: 0.65–0.84) were significant risk factors for 30-day postoperative mortality ( Table 3 ). The final model showed a Nagelkerke R ^2^ of 27.1%. ROC analysis revealed an AUC of 0.78, indicating a moderate discriminatory performance of the model ( Fig. 1 ).

**Table 2:** Univariable logistic regression for 30-day mortality, necrotizing enterocolitis-totalis and mortality without necrotizing enterocolitis-totalis

Variable	30-day mortality, *N* = 137		NEC-T, *N* = 75		Mortality without NEC-T, *N* = 62	
	OR (95% CI)	*p* -Value	OR (95% CI)	*p* -Value	OR (95% CI)	*p* -Value
Male sex	1.86 (1.20–2.87)	0.005	1.79 (1.04–3.08)	0.036	1.50 (0.84–2.66)	0.170
Birthweight per 100 g	0.91 (0.86–0.96)	<0.001	0.91 (0.85–0.97)	0.006	0.94 (0.88–1.01)	0.081
Cardiovascular support between NEC and surgery a	3.14 (2.04–4.83)	<0.001	2.58 (1.54–4.33)	<0.001	2.32 (1.33–4.04)	0.003
Portal venous gas	1.76 (1.11–2.78)	0.016	2.23 (1.31–3.79)	0.003	0.98 (0.53–1.81)	0.941
Time between diagnosis and surgery in days	0.75 (0.66–0.85)	<0.001	0.81 (0.70–0.93)	0.002	0.76 (0.64–0.91)	0.002
Pneumatosis intestinalis	1.19 (0.73–1.96)	0.489	1.26 (0.68–2.34)	0.468	1.04 (0.55–1.99)	0.901
Multiple birth	1.15 (0.74–1.80)	0.542	1.20 (0.70–2.05)	0.509	1.03 (0.57–1.85)	0.930
Cardiac anomaly	0.59 (0.23–1.51)	0.273	1.09 (0.40–3.01)	0.864	0.22 (0.03–1.62)	0.136
Pneumoperitoneum	0.93 (0.62–1.40)	0.725	0.74 (0.45–1.22)	0.240	1.25 (0.73–2.15)	0.420
Cesarean section	1.03 (0.68–1.56)	0.904	0.96 (0.58–1.58)	0.867	1.10 (0.64–1.89)	0.735

Abbreviations: NEC, necrotizing enterocolitis; NEC-T, necrotizing enterocolitis-totalis; OR, odds ratio.

a Four missings.

**Table 3:** Multivariable logistic regression analysis of 30-day mortality, necrotizing enterocolitis-totalis and mortality without necrotizing enterocolitis-totalis

Variable	OR	95% CI	*p* -Value
30-day mortality			
Male sex	2.53	1.54–4.16	<0.001
Birthweight per 100 g	0.91	0.86–0.96	0.002
Cardiovascular support between NEC and surgery a	3.26	2.02–5.24	<0.001
Time between diagnosis and surgery in days	0.74	0.65–0.84	<0.001
Portal venous gas	1.89	1.11–3.20	0.019
NEC-T			
Male sex	2.00	1.12–3.57	0.019
Birthweight per 100 g	0.91	0.84–0.98	0.011
Cardiovascular support between NEC and surgery a	2.16	1.24–3.76	0.007
Time between diagnosis and surgery in days	0.69	0.57–0.83	<0.001
Portal venous gas	2.42	1.35–4.35	0.003
Mortality without NEC-T			
Cardiovascular support between NEC and surgery a	2.33	1.33–4.09	0.003
Time between diagnosis and surgery in days	0.77	0.64–0.91	0.002

Abbreviations: CI, confidence interval; NEC, necrotizing enterocolitis; NEC-T, necrotizing enterocolitis-totalis; OR, odds ratio.

a Four missings.

### Risk Factors for Mortality in Neonates with Necrotizing Enterocolitis Totalis

**Fig. 2:**
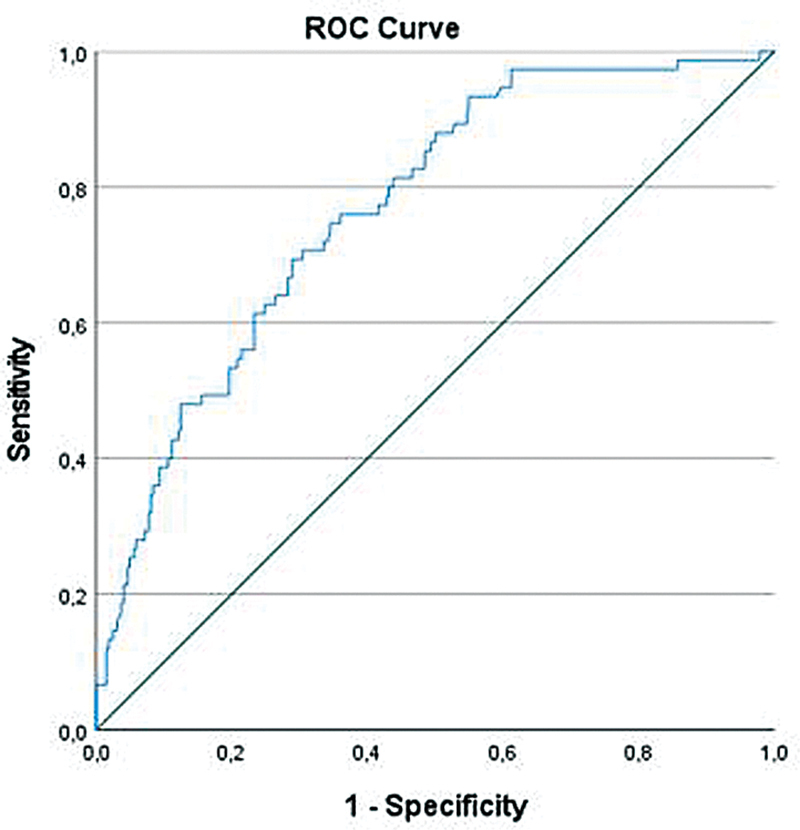
ROC curve of predicted probabilities of NEC totalis. NEC, necrotizing enterocolitis; ROC, receiver operating characteristics. Area under the curve: 0.77.

Univariable logistic regression for NEC-T showed that male sex, birthweight, need for cardiovascular support between NEC onset and surgery, reported PVG on radiological imaging, and time from onset to surgery (in days) were significantly related to mortality in neonates with NEC-T and were subsequently included in the multivariable analysis ( Table 2 ). Multivariable logistic regression showed that male sex (OR: 2.00; 95% CI: 1.12–3.57), birthweight (OR: 0.91; 95% CI: 0.84–0.98, per 100 g), signs of PVG on radiological imaging as mentioned in the reports (OR: 2.42; 95% CI: 1.35–4.35), need for cardiovascular support between NEC onset and surgery (OR: 2.16; 95% CI: 1.24–3.76), and time (in days) between onset and surgery (OR: 0.77; 95% CI: 0.64–0.91) were significant risk factors for mortality with NEC-T ( Table 3 ). The final model showed a Nagelkerke R ^2^ of 21.3% and an AUC of 0.77, indicating a moderate discriminatory performance of the model ( Fig. 2 ).

### Risk Factors for Mortality in Neonates without Necrotizing Enterocolitis Totalis

**Fig. 3:**
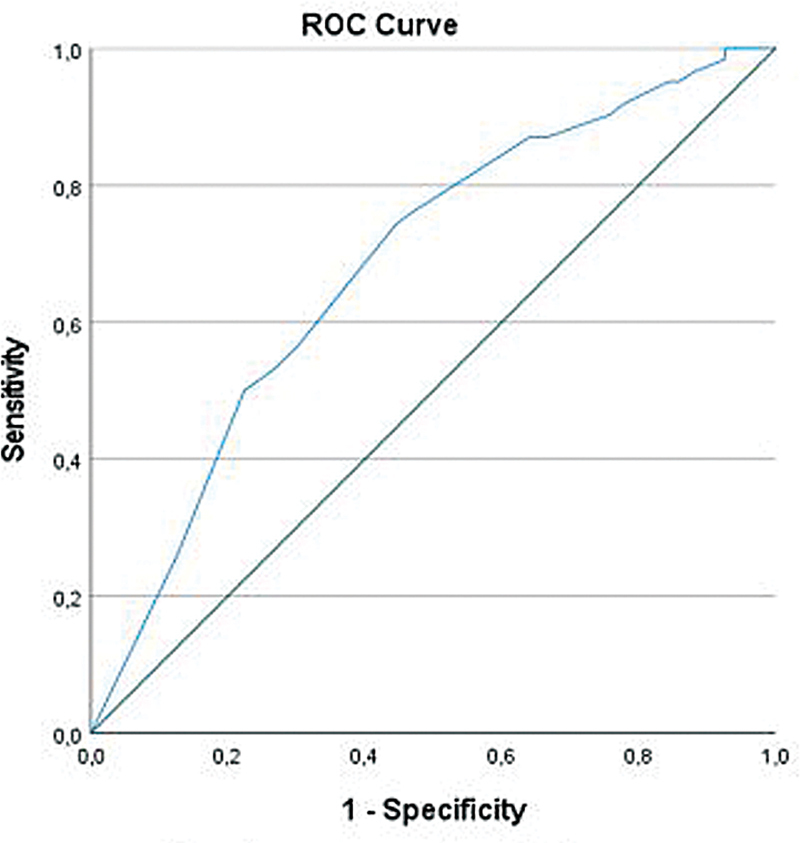
ROC curve of predicted probabilities of mortality without NEC totalis. NEC, necrotizing enterocolitis; ROC, receiver operating characteristics. Area under the curve: 0.603.

For the patients who deceased within 30 days after surgery without NEC-T, univariable logistic regression analysis showed that need for cardiovascular support between NEC onset and surgery (OR: 2.33 95% CI: 1.33–4.09) ( Table 2 ) and time from onset to surgery (OR: 0.77 95% CI: 0.64–0.91) were significant risk factors for mortality without NEC-T. For this model, a Nagelkerke R ^2^ of 9.6% was found. ROC analysis showed an AUC of 0.68, indicating a poor discriminatory performance of the model ( Fig. 3 ).

## Discussion

In this cohort of 401 preterm born infants surgically treated for NEC, the 30-day mortality rate was 34.2%, with NEC-T contributing significantly. Preoperative risk factors for 30-day mortality included male sex, lower birthweight, reported presence of PVG on abdominal radiography, the preoperative need for cardiovascular support, and shorter time between disease onset and surgery. Similar risk factors were identified for NEC-T. In patients without NEC-T, mortality risk was associated with preoperative cardiovascular support and shorter interval from onset to surgery.

The current study's findings align with existing literature reporting 30-day mortality rates of 23 to 39%, 2 20 21 with higher rates among lower birthweight infants. 2 Calvert et al 22 identified GA and birthweight <1,000 g as significant predictors of mortality (both 30-day and 1-year) in surgical NEC. The present study adds male sex, PVG on radiography (reports), preoperative cardiovascular support, and a shorter time interval to surgery as new risk factors. The association between male sex and worse surgical outcomes is consistent with findings of increased neonatal complications in males. 23 24 25 The underlying mechanisms are still partially understood and likely involve an interplay between genetics, hormones, oxidative stress, and immunology, requiring further study. 26 27

While previous studies have explored PVG's association with surgical NEC and mortality, the present study uniquely identifies reported PVG as a risk factor for 30-day mortality. 28 29 30 31 PVG may indicate a more advanced disease stage, given its pathophysiology. Pneumatosis intestinalis results from bacterial translocation and hydrogen gas formation in the bowel wall. This gas can embolize into the mesenteric veins toward the portal venous system, causing in PVG, which likely indicates severe NEC. The role of PVG in surgical decision-making warrants further investigation.

The preoperative need for cardiovascular support was associated with a 3-fold increase in 30-day mortality, reflecting severe illness with hemodynamic instability. Bonasso et al 32 similarly found higher mortality in NEC patients requiring cardiovascular support. Their study of 901 surgically treated NEC patients showed a 19% mortality rate, with those with preoperative cardiovascular support more likely to die postoperatively. These findings underscore the gravity of preoperative cardiovascular instability and its impact on outcomes.

More than half of the patients underwent surgery within 1 day of disease onset. Interestingly, our data showed the highest mortality in those who were operated the soonest after NEC onset (<1 day). This may appear counter-intuitive, as one would expect higher mortality in those with a longer interval to surgery. However, considering the availability of surgery around the clock, the most likely explanation for finding the opposite, is that the time to surgery reflects the severity of the patients' condition. A more expectative approach was followed in patients with a relatively stable condition, whereas those with severe, rapidly deteriorating NEC underwent surgery as soon as possible. Thus, the time to surgery indicates clinical severity rather than surgical delay. A post hoc data exploration substantiated this hypothesis: a shorter time interval was associated with pneumoperitoneum on abdominal radiography (data not shown). Therefore, we conclude that our observational data should be interpreted with great caution and should not lead to the erroneous conclusion that postponing surgery improves survival.

NEC-T affected 18.7% of our cohort and accounted for over half of the deaths, underscoring its rapid onset and fatal outcomes. Comparable studies report similar incidences, ranging from 11 to 21%. 4 16 33 34 35 Estimating the incidence of NEC-T remains difficult due to variations in definitions. Given its significant impact on mortality in our cohort, it is understandable that risk factors for 30-day mortality overlap with those for NEC-T. To our knowledge, the current study represents the largest investigation into risk factors for NEC-T to date. Previous research, limited by small sample size ( *n* = 39), failed to identify significant associations, underscoring the need for larger, prospective studies to establish definitive predictive factors. 33 A comprehensive understanding of the pathophysiology of NEC-T is essential for developing effective diagnostic and therapeutic approaches aimed at prevention and improved treatment outcomes. For instance, advantages in short bowel rehabilitation and intestinal transplantation may lead to improved and meaningful long-term outcomes, potentially offering more treatment options for patients with extensive bowel necrosis and reducing mortality. 36 37

Further analysis of mortality among patients without NEC-T revealed only preoperative cardiovascular support and time (in days) from NEC onset to surgery emerged as significant risk factors. Mortality in this group was attributed to ongoing sepsis, multiorgan failure, neurological deterioration, or persistent NEC. In most of these patients, cardiovascular support was continued postoperatively, suggesting that severe pre- and postoperative hemodynamic instability contributed to mortality. This may explain why preoperative cardiovascular support and shorter time to surgery emerged as significant risk factors for mortality. Interestingly, male sex, lower birthweight, and PVG were not identified as risk factors in this subgroup, which contrasts with findings in NEC-T cases. It is plausible that this is either due to limited power or because these risk factors are more closely linked to NEC-T and therefore did not influence mortality in the absence of NEC-T.

## Limitations and Strengths

The main limitation of this study is its retrospective design, which only allows for the investigation of associations rather than causations. Retrospective research is subject to missing or underreported data, potentially masking important risk factors such as antenatal medication, cardiac anomalies, and pulmonary condition. In this study, we relied on the radiological reports, as these were available at the time of clinical decision-making. Despite pediatric radiologists being available 24/7 in each center, misinterpretation of radiological signs may have occurred. To enhance generalizability to real-life clinical settings, we decided not to reassess the original images in retrospect. Further exploration of the predictive value of radiological signs and their role in decision-making in surgical NEC requires further study in larger cohorts. Also of note, we investigated only the surgically treated NEC patients, as risk factors for medical NEC have been extensively studied. Understanding surgical outcomes, especially for NEC-T, is crucial for improving management and counseling. The incidence of surgical and medical NEC and their outcomes on mortality have previously been described in a Dutch single-center study. 38 This approach automatically excluded patients with medically treated severe NEC who were deemed too unstable for surgery and subsequently deceased without surgery. This may lead to underreporting of NEC-T cases, not only in this population but also in other cohorts. In order to accurately identify global risk factors for NEC-T, a clear standardized definition should first be established.

This study's multicenter setting presents both limitations and strengths. Variations in clinical judgement, decision-making, and patient management due to the lack of universal guidelines for treating (surgical) NEC, as well as differences in strategies among surgeons within a center, may influence study outcomes. Despite these variations, the multicenter design strengthened our study by including a large cohort from three major NICU centers in the Netherlands, facilitating cross-validation. Although this allowed for statistical models to identify multiple unique risk factors, statistical power was still too limited to explore for example demographic or socioeconomic factors and biochemical measures. Also, variation in treatment regarding antibiotic and probiotic usage, nutritional and respiratory support, as well as surgical approaches require further study. Further research is necessary to validate our findings, to further explore relevant factors and to develop and validate prediction tools for clinical decision-making and parental counseling. These can help clinicians make informed decisions regarding monitoring, interventions, and surgical planning. For example, recognizing that an infant has a higher risk of poor outcomes may prompt clinicians to opt for earlier surgical intervention or more intensive preoperative care. They can guide personalized counseling based on the infant's individual risk profile and prepare parents emotionally for potential outcomes.

## Conclusion

This study found that male sex, lower birthweight, PVG on abdominal radiography, need for cardiovascular support, and an interval of <1 day between NEC onset and surgery were risk factors for both 30-day mortality in infants with surgical NEC and the development of NEC-T. These findings contribute valuable insights to the existing relevant literature. Specifically, for short-term mortality without NEC-T, the need for cardiovascular support and an interval of <1 day between NEC onset and surgery emerged as significant risk factors. External validation is required to confirm these results. Insight into these preoperative risk factors may serve parental counseling.
