# Supplementary material for: Risk Factors for 30-day Mortality in Patients with Surgically Treated Necrotizing Enterocolitis: A Multicenter Retrospective Cohort Study
Source: Eur J Pediatr Surg. 2025 Mar 21;35(4):332–40. doi: 10.1055/a-2536-4757 (PMC12245516; doi:10.1055/a-2536-4757)
Supplement: Supplementary file 1 — Supplementary Material [file 10-1055-a-2536-4757-s2025017178oa.pdf]

Supplementary Appendix A Intraoperative characteristics of mortality without necrotizing enterocolitis-totalis group

**Intraoperative characteristics**[a]		**Total** ***N*** **= 62**
Perforation during surgery		48 (77.4)
Location of perforation	Jejunum	6 (12.5)
	Ileum	17 (35.4)
	Small bowel (not specified)	3 (6.3)
	Colon	15 (31.3)
	Small bowel and colon	6 (12.5)
	Unclear	1 (2.1)
Ascites during surgery		42 (67.7)
Small bowel resection		37 (59.7)
Length of resected small bowel, cm		12.0 (5.0–25.0)
Colon resection		15 (24.2)
Length of resected colon, cm		9.5 (4.0–12.3)

[a]Data presented as number (%) or median (interquartile range).

 © 2025. The Author(s).
